# Effect of Extrusion Ratio on Microstructure and Mechanical Properties of 7A85 Aluminum Alloy

**DOI:** 10.3390/ma19204403

**Published:** 2026-10-20

**Authors:** Zhengbin Ma, Wenli Gao

**Affiliations:** 1 School of Materials Science and Engineering, Hunan University, Changsha 410082, China; 2 Beijing Hangxing Machinery Co., Ltd., Beijing 100013, China

**Keywords:** 7A85 aluminum alloy, extrusion ratio, dynamic recrystallization, mechanical properties, EBSD, substructure strengthening

## Abstract

The extrusion ratio strongly influences the deformation behavior and final properties of aluminum alloy extrusions. In this study, the effects of extrusion ratio (13, 16, 20, and 26) on the microstructure and mechanical properties of 7A85 aluminum alloy were investigated by tensile testing, hardness and electrical conductivity measurements, EBSD characterization, and DEFORM-3D finite element simulation. The results show that the sample with an extrusion ratio of 13 has the finest recrystallized grains (5.73 μm) and the highest average misorientation angle (26.25°), indicating a relatively high degree of recrystallization. In contrast, although the sample with an extrusion ratio of 20 exhibited a mixed grain structure (a mixture of fine and coarse grains) and incomplete recrystallization, it achieved the highest as-extruded tensile strength (502 MPa). The strength degradation of the sample with an extrusion ratio of 26 is due to excessive recovery and grain coarsening. After T6 heat treatment, the strength difference between samples with different extrusion ratios was basically eliminated (~690–697 MPa). Combined with the simulation results under different extrusion ratios, the temperature field and material flow field in the extrusion process were analyzed, and the deformation and recrystallization mechanisms were deeply understood. These results indicate that the intermediate extrusion ratio can provide the best balance for the as-extruded material’s strength, and the T6 aging response is relatively insensitive to the extrusion ratio. However, fine and uniform recrystallized grains are more conducive to a stable aging response. This work provides guidance for optimizing the extrusion process parameters of 7A85 aluminum alloy.

## 1. Introduction

Aluminum alloys are widely used in aerospace, automotive, and other transportation sectors owing to their comprehensive advantages of high specific strength, excellent corrosion resistance, superior formability, and outstanding recyclability [1,2,3,4]. In the context of sustainable manufacturing and the circular economy, the recyclability of aluminum alloys has become a vital engineering advantage [5]. In addition to their large-scale application as integral load-bearing structural materials, high-strength 7xxx series aluminum alloys can also serve as the matrix of metal matrix composites (MMCs). The introduction of ceramic particles, reinforcing fibers, carbon nanofillers, and other reinforcing phases can effectively improve the stiffness, wear resistance, and high-temperature service stability of the materials [6,7]. However, whether used as monolithic structural materials or as composite matrices, the final performance of aluminum alloys is governed by their microstructure. Hot extrusion, as a key plastic forming process for 7xxx series aluminum alloys, directly determines the microstructural evolution and service performance of the material through its processing parameters.

The growing demand for lightweight and high-performance structural materials in the aerospace and high-end automotive industries has promoted in-depth research on the coupling relationship among the forming process, microstructure, and mechanical properties of high-strength aluminum alloys. Among commercial high-strength wrought aluminum alloys, the Al-Zn-Mg-Cu 7xxx series alloys exhibit the highest strength and have been widely verified and applied as core load-bearing structural materials under severe aerospace service conditions [8,9,10]. Among them, 7085 aluminum alloy is extensively adopted for the main load-bearing structural components of large aircraft due to its excellent hardenability, serving as a typical high-grade aerospace structural aluminum alloy. Based on the composition system of 7085 alloy, 7A85 aluminum alloy is a novel high-strength and high-toughness aluminum alloy independently developed in China through precise composition regulation and optimization of extrusion and heat treatment processes. It effectively improves the fracture toughness and reduces the quenching sensitivity, which better meets the forming and service requirements of high-end complex aerospace structural parts [11,12,13]. The extrusion ratio, as a core parameter of the extrusion process, directly affects the degree of deformation, temperature rise, dynamic recovery, and recrystallization behavior, thereby determining the microstructure and mechanical properties. Accordingly, systematic research on the recrystallization evolution behavior of 7A85 aluminum alloy during extrusion forming is of great theoretical significance and engineering value for process optimization, microstructure regulation and comprehensive mechanical property improvement.

In recent years, the hot deformation behavior and microstructure evolution of 7A85 and related 7085 aluminum alloys have attracted considerable research attention. Zhang et al. [14] investigated the high-temperature rheological behavior and microstructure evolution of as-extruded 7A85 aluminum alloy through unidirectional isothermal compression tests, revealing that the dynamic softening mechanism transforms from dynamic recovery to dynamic recrystallization with increasing deformation temperature and decreasing strain rate. The dislocation density model established for 7A85 aluminum alloy during thermal deformation further confirmed that dynamic recovery and dynamic recrystallization are the dominant microstructure evolution mechanisms, with the temperature range of 350–400 °C identified as the transition zone from dynamic recovery to dynamic recrystallization. Mei et al. [15] conducted a comprehensive study on the hot deformation behavior of 7085 aluminum alloy during backward extrusion, demonstrating that dynamic recrystallization occurs during hot compression and that the peak stress increases with increasing strain rate and decreasing deformation temperature. Wang et al. [16] developed a constitutive model and processing maps for 7085 aluminum alloy, showing that while the true stress–strain curves predominantly exhibit dynamic recovery characteristics, the softening mechanism shifts toward dynamic recrystallization at higher temperatures and lower strain rates.

Extrusion is a primary manufacturing process for aluminum alloy profiles. The extrusion ratio, as a core parameter, directly governs the degree of deformation and resultant mechanical properties. Studies on Al-Zn-Mg-Cu alloys have shown that increasing the extrusion ratio generally refines grains and improves strength, but excessive ratios lead to complete dynamic recrystallization and coarse precipitation, causing property decline [17,18,19]. Xu et al. [20] further demonstrated that recrystallization behavior is strongly dependent on stored energy level and heating conditions. Although current research has made great progress, the systematic study of the effect of extrusion ratio on the microstructure evolution of 7A85 aluminum alloy is still limited, especially regarding grain size distribution, misorientation characteristics, and texture development. In addition, the coupling relationship between the microstructure inhomogeneity caused by the extrusion ratio and the mechanical properties of the extruded and heat-treated states has not been fully elucidated.

In this study, the effects of extrusion ratio (13, 16, 20, and 26) on the mechanical properties, electrical conductivity, and microstructure evolution (grain size, misorientation, and texture) of 7A85 aluminum alloy extrusions were systematically investigated. Combined with DEFORM-3D finite element numerical simulation, the underlying mechanisms governing extrusion-ratio-dependent microstructure evolution were revealed, providing a reference for the optimization of extrusion process parameters for this alloy.

## 2. Materials and Methods

### 2.1. Experimental Materials

The experimental material was a 7A85 aluminum alloy billet. After homogenization and surface peeling, the billet was extruded in a container with an inner diameter of 180 mm. Extrusion experiments were conducted using an 18 MN direct extrusion press (SMS Meer, Germany). Prior to extrusion, both the container and the dies were preheated to 400 °C. Extrusion was carried out at a temperature of 390 °C with a ram speed of 1 mm/s. Four extrusion ratios of 13, 16, 20, and 26 were employed to obtain the as-extruded specimens.

After extrusion, the extrudates were subjected to solution treatment at 476 °C for 3 h, followed by water quenching to room temperature and subsequent artificial aging at 120 °C for 24 h to achieve the T6 temper. Finally, specimens for subsequent performance testing were sectioned from both the as-extruded and T6-tempered materials parallel to the extrusion direction.

### 2.2. Finite Element (FE) Model

**Figure 1:**
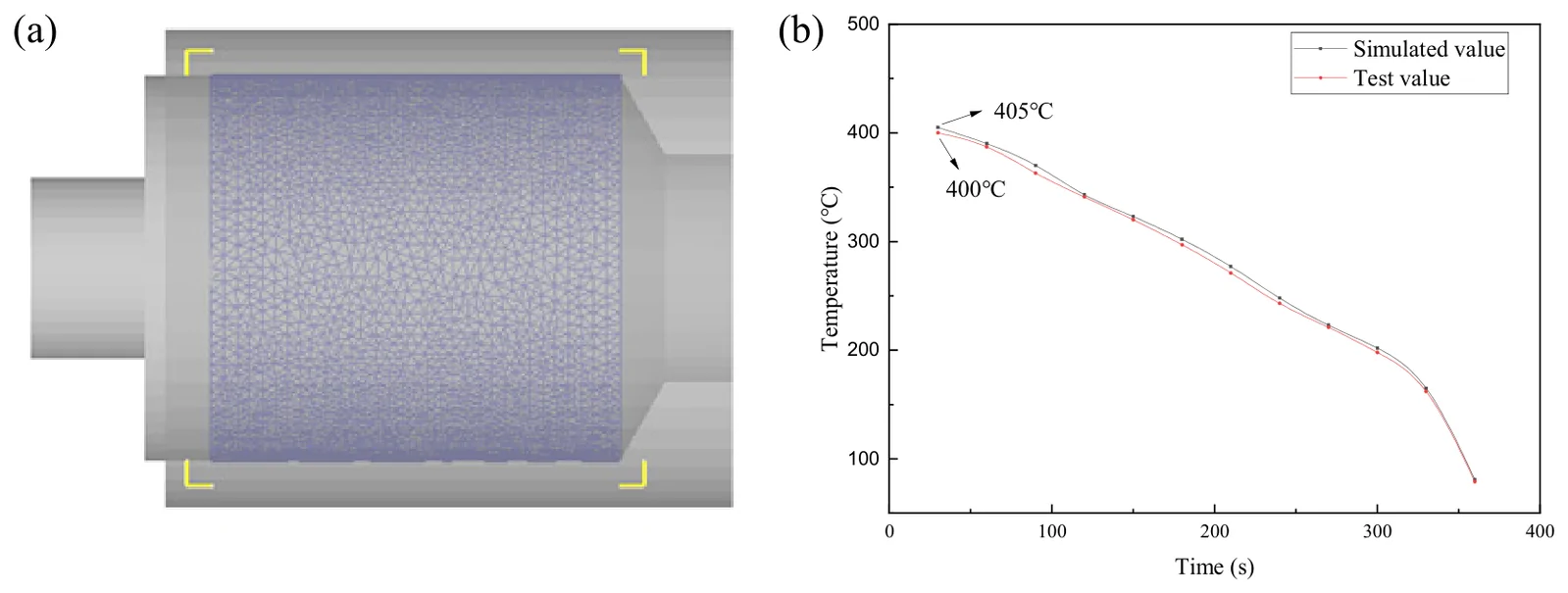
Finite element analysis model: (**a**) mesh generation; (**b**) temperature variation curves.

In order to analyze the temperature field and metal flow behavior of 7A85 aluminum alloy during hot extrusion, a three-dimensional thermo-mechanical coupling finite element model is established using DEFORM-3D (V14.1) finite element analysis software. The model mainly consists of three parts: an extrusion container, ingot, and extrusion ram. Among them, the ingot is defined as the deformable body, while the extrusion container and extrusion ram are treated as rigid bodies. The ingot material is set as 7A85 aluminum alloy, and the overall structure of the model is shown in Figure 1a. In this model, the ingot dimensions are Φ170 mm × 180 mm, and the die exit diameter is selected as Φ40 mm, corresponding to an extrusion ratio of 20. The initial extrusion temperature is set to 390 °C, the extrusion ram speed is 1.0 mm/s, and the total extrusion time is 180 s. The billet is discretized with four-node tetrahedral elements. The initial finite element model of the workpiece consisted of 20,486 nodes and 97,146 elements. To maintain satisfactory mesh quality under large deformation, the adaptive mesh remeshing capability in DEFORM-3D was enabled during the simulation. The upper and lower dies were assigned as rigid bodies and only served to define contact boundaries and geometric constraints.

The plastic flow stress is described by a power-law function [21]:

$$\bar{\sigma }=c{\bar{\varepsilon }}^{n}{\overset{\cdot }{\bar{\varepsilon }}}^{m}+y$$

where *c* = 95, *n* = 0, *m* = 0.14, *y* = 0. This model can preliminarily characterize the strain rate sensitivity of 7A85 aluminum alloy during hot extrusion. In the simulation, the conversion coefficient from plastic deformation work to heat energy is set to 0.9 to account for heat accumulation induced by plastic deformation. In the present model, the contact heat transfer coefficient between the workpiece and dies is defined as 0. Accordingly, the simulation results mainly reflect the influences of heat generated by plastic deformation and friction on the temperature field.

To validate the accuracy of the finite element simulation in this study, a handheld contact thermocouple was used to continuously measure the temperature at the tip of the extrudate at intervals of 30 s, and the measured results were compared with the simulation results. Figure 1b presents the temperature–time curves obtained from both the experimental measurement and simulation. During hot extrusion, the experimental and simulated temperature variations exhibited essentially the same trend, both gradually decreasing. The peak temperature measured experimentally was 400 °C, while that predicted by simulation was 405 °C, with an error of only 1.3% between them, confirming the reliability of the established finite element model.

### 2.3. Experimental Methods

Tensile test specimens with a diameter of 5 mm and a gauge length of 25 mm were machined from the center of the extruded bars. Tensile tests were conducted on a universal testing machine at an initial strain rate of 2.5 × 10^−4^ s^−1^, and the average values from three specimens were taken for each condition. Electrical conductivity measurements were performed at room temperature using an eddy current conductivity meter. The specimen surfaces were polished to ensure freedom from oil contamination and oxide films. Five test points were evenly selected on each specimen, and the average value was taken. Brinell hardness tests were carried out with a test force of 1000 N and a holding time of 30 s. The specimen surfaces were polished to remove oxidation layers and work-hardened layers. At least five hardness readings were taken at different positions on each specimen, and the average value was calculated.

For EBSD analysis, the specimens were mechanically ground and polished, followed by vibratory polishing to remove surface residual stresses. Observations were performed using a TESCAN field-emission scanning electron microscope (S8000, Czechia) with a scan step size of 0.8 μm and an accelerating voltage of 20 kV. The acquired data were subsequently post-processed using OIM Analysis software (TSL 7.3).

## 3. Results

### 3.1. Effect of Extrusion Ratio on Tensile Properties

**Figure 2:**
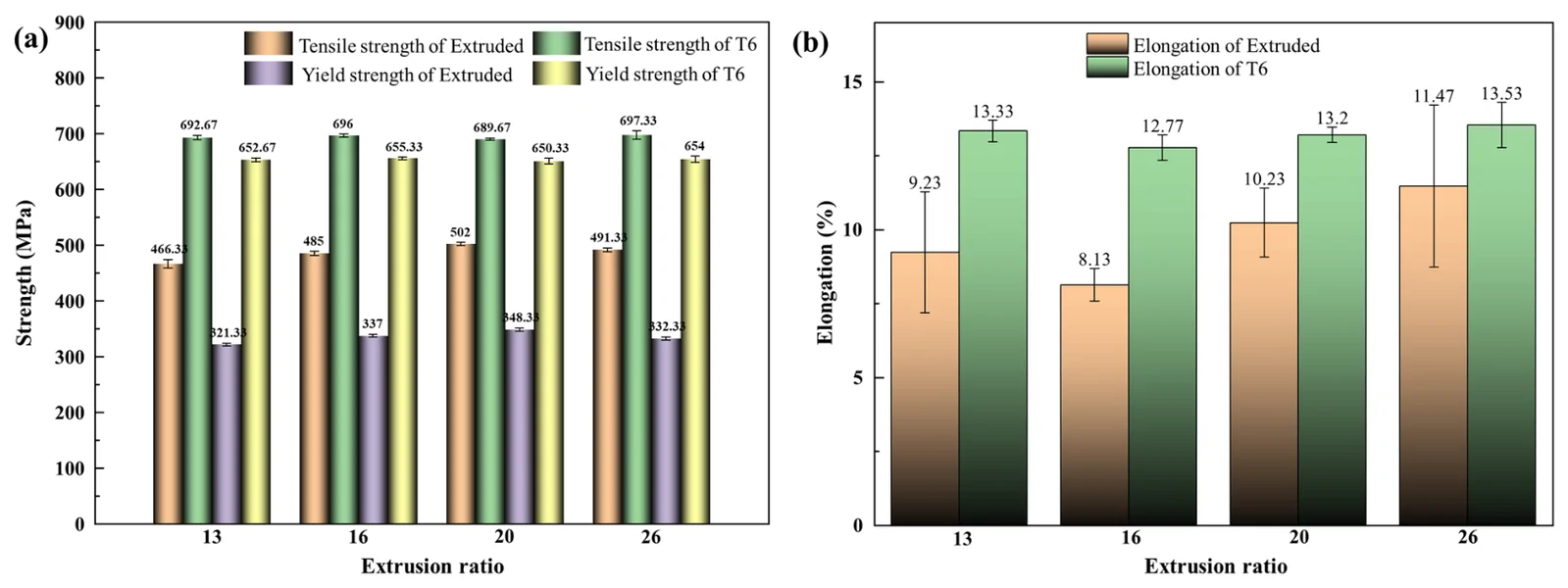
Test results of mechanical properties under different states: (**a**) the tensile and yield strength; (**b**) the elongation.

The mechanical properties of the as-extruded alloy exhibited a different trend compared to those of the T6-treated alloy as the extrusion ratio increased. As shown in Figure 2a, the tensile strength of the as-extruded alloy initially increased and then decreased with increasing extrusion ratio. When the extrusion ratio rose from 13 to 16, the tensile strength increased from 466.33 MPa to 485 MPa. It reached a peak value of 502 MPa at an extrusion ratio of 20. However, when the extrusion ratio further increased to 26, the tensile strength dropped to 491.33 MPa. The yield strength of the as-extruded alloy showed a similar trend, reaching a maximum value of 348.33 MPa at an extrusion ratio of 20. The mechanical properties of the T6-treated alloy were different from those of the as-extruded alloy as the extrusion ratio increased. The tensile strength and yield strength of the T6-treated alloy remained stable within the ranges of 690 to 697 MPa and 650 to 655 MPa, respectively.

The elongation results shown in Figure 2b indicated that for the as-extruded alloy, the elongation decreased initially and then increased as the extrusion ratio increased. When the extrusion ratio was 13, the elongation of the as-extruded alloy was 9.23%. When the extrusion ratio was 16, it dropped to the lowest value of 8.13%. When the extrusion ratio increased to 20 and 26, it rose to 10.23% and 11.47%, respectively. This trend suggested that under an appropriate extrusion ratio (such as 20), the as-extruded alloy could achieve a better strength-plasticity match. However, when the extrusion ratio was too large (such as 26), although the elongation further increased, the strength decreased. For the T6-treated alloy, the change in extrusion ratio had a relatively small effect on the elongation, and the elongation remained stable at 12.77% to 13.53%. The difference in performance between the as-extruded and T6-treated alloys indicated that heat treatment could significantly reduce the influence of the extrusion structure on the final performance. After T6 treatment, the differences in strength and elongation between samples with different extrusion ratios were significantly reduced, indicating that the mechanical properties of the aged state were relatively insensitive to the extrusion ratio.

### 3.2. Effect of Extrusion Ratio on Hardness

**Figure 3:**
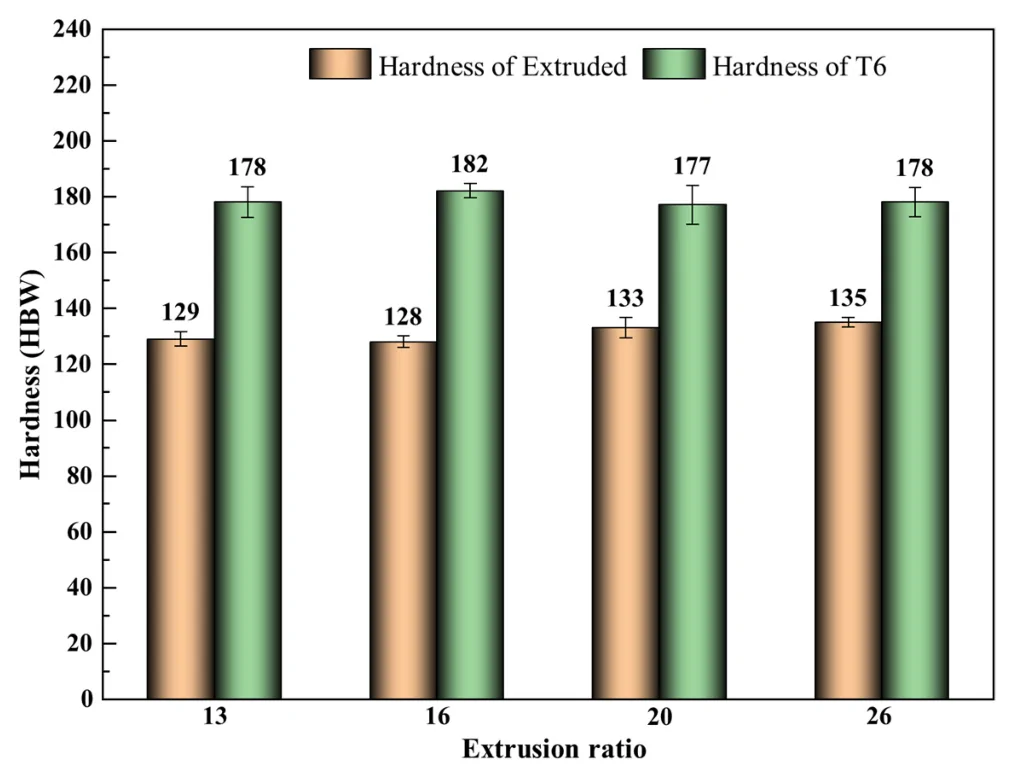
Hardness test results of materials under different states.

Figure 3 shows the hardness results of the alloys with different extrusion ratios in as-extruded and T6-treated states. The hardness in the extruded state ranged from 128 to 135 HBW and generally increased with the increase in the extrusion ratio, but it was not a monotonically increasing trend. The hardness at an extrusion ratio of 16 (128 HBW) was slightly lower than at 13 (129 HBW). When the extrusion ratio increased to 20 and 26, the hardness reached 133 and 135 HBW, respectively. It should be noted that the change in hardness was not completely consistent with the change in tensile strength. The tensile strength reached its peak at an extrusion ratio of 20 and decreased at 26, while the hardness remained the highest at 26. This was mainly because hardness reflected the resistance to local plastic deformation and was less sensitive to coarse grains and microscopic defects than macroscopic tensile properties. After T6 treatment, the hardness of alloys with different extrusion ratios was 177 to 182 HBW, with a range difference of only 5 HBW, indicating that the extrusion ratio had a relatively small influence on the hardness of the T6 state. The differences in hardness and strength after T6 treatment had significantly decreased, indicating that the heat treatment significantly weakened the influence of the extrusion structure on the final mechanical properties.

The change in hardness was influenced by various factors such as deformation degree, dislocation density, grain size, and second phases. During hot extrusion, an increase in the extrusion ratio led to an increase in the strain, and under conditions where dynamic recovery was insufficient, dislocation density and the number of substructures may increase, thereby enhancing the extruded hardness through dislocation/substructure strengthening. However, an increase in the extrusion ratio also resulted in an increase in the deformation temperature, promoting dynamic recovery and consuming some dislocations and substructures. In this study, the as-extruded hardness increased from 129 HBW at an extrusion ratio of 13 to 135 HBW at 26, but the tensile strength decreased at an extrusion ratio of 26. This indicated that the responses of hardness and strength to the change in microstructure were not completely consistent. Hardness was less sensitive to coarse grains and microscopic defects. After T6 heat treatment, precipitation strengthening became the dominant factor, and the hardness differences among samples with different extrusion ratios significantly decreased.

### 3.3. Effect of Extrusion Ratio on Electrical Conductivity

**Figure 4:**
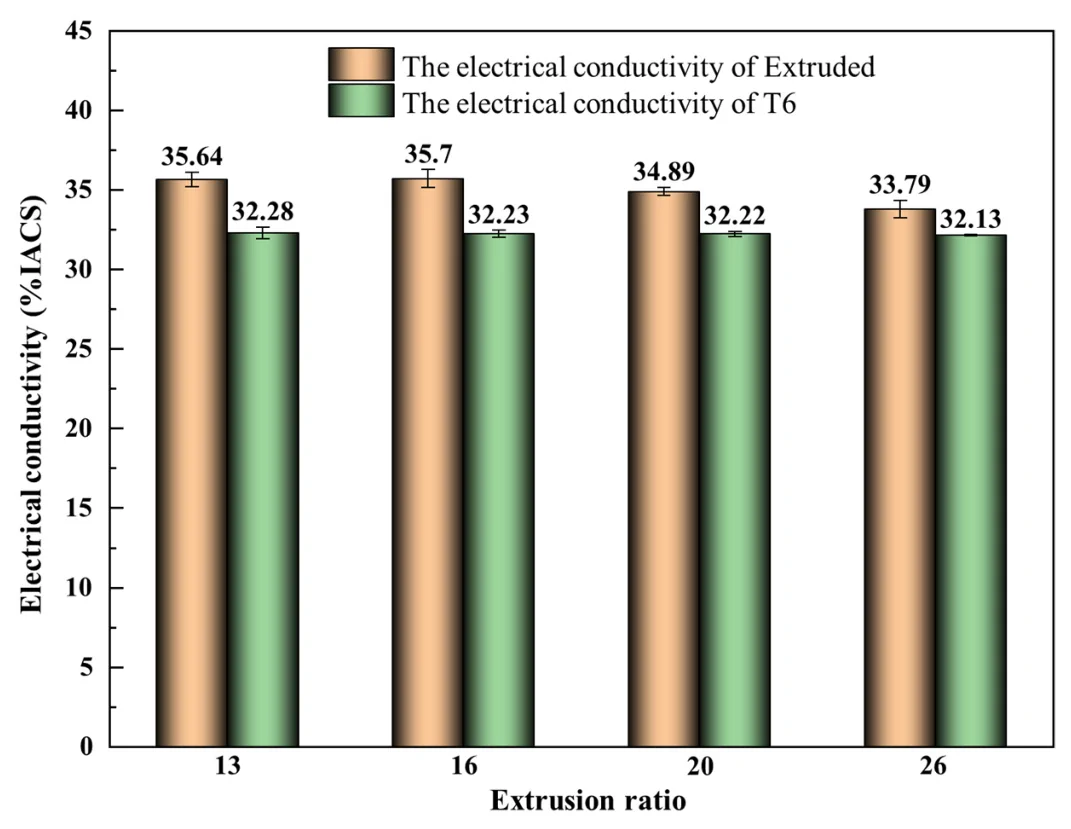
The electrical conductivity test results of materials under different states.

Figure 4 showed the electrical conductivity changes of alloys with different extrusion ratios in the extruded state and T6 state. For the as-extruded alloys, the electrical conductivity decreased significantly with the increase in extrusion ratio. It dropped from 35.64% IACS at an extrusion ratio of 13 to 33.79% IACS at 26, with a decrease of 1.85% IACS. After T6 heat treatment, the electrical conductivity decreased overall, but the variation range was very small, dropping from 32.28% IACS to 32.13% IACS, with a decrease of 0.15% IACS. By comparison, the electrical conductivity in the extruded state was more sensitive to the extrusion ratio, while the electrical conductivity in the T6 state remained basically stable under different extrusion ratios. The electrical conductivity reflected the solubility and precipitation state of the matrix to a certain extent. Under the extruded state, the deformation temperature rose and the dynamic recovery degrees caused by different extrusion ratios were different, which may lead to the precipitation of solute atoms and differences in the solubility of the matrix, thus resulting in more obvious changes in conductivity. After T6 treatment, the electrical conductivity differences of samples with different extrusion ratios significantly decreased, which may be related to the solubility and aging precipitation processes.

## 4. Discussion

### 4.1. Analysis of Extrusion Forming Process

**Figure 5:**
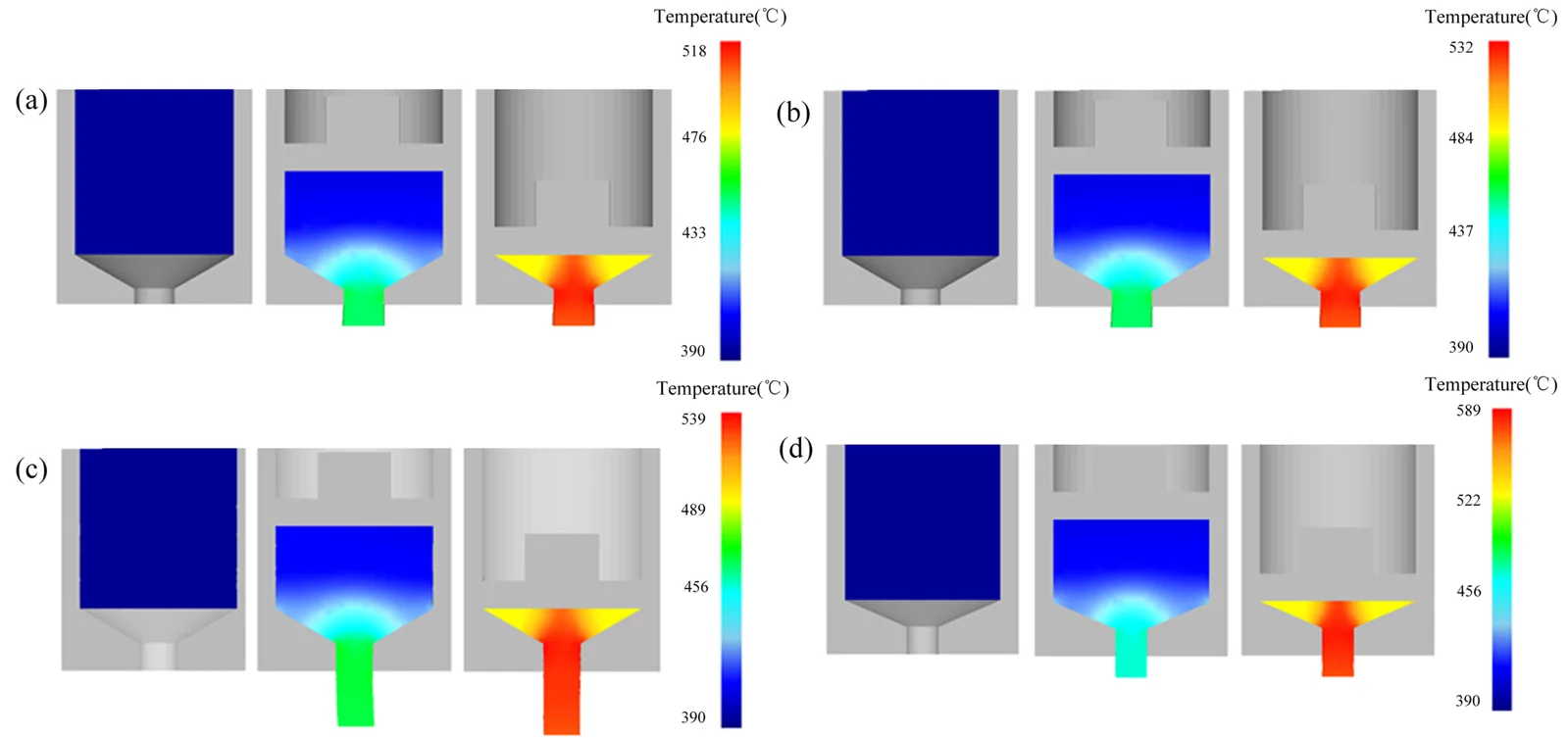
Temperature field during extrusion at 0 s/90 s/180 s with different extrusion ratios: (**a**) 13; (**b**) 16; (**c**) 20; (**d**) 26.

Figure 5a–d presents the temperature field distributions during hot extrusion of 7A85 aluminum alloy at 0 s, 90 s and 180 s under extrusion ratios of 13, 16, 20 and 26, respectively. At the initial moment of 0 s, the billet temperature is uniform at approximately 390 °C, and no localized temperature rise is observed for any extrusion ratios. At 90 s, plastic deformation is concentrated in the die orifice deformation zone. Plastic work and interfacial friction generate substantial deformation heat, leading to obvious local temperature elevation in the shear band region. As the extrusion ratio increases, the magnitude of this temperature rise gradually increases. At the steady-state extrusion stage of 180 s, the maximum temperature is located at the extrudate near the die exit. The peak temperature is 518 °C at the extrusion ratio of 13, which increases to 532 °C and 539 °C for extrusion ratios of 16 and 20, respectively. The highest peak temperature of 589 °C is obtained when the extrusion ratio reaches 26.

A higher extrusion ratio imposes larger effective strain and more intensive shear deformation, producing more plastic dissipation heat and thus elevating peak temperature. Meanwhile, the high-temperature region expands with increased extrusion ratio. For low extrusion ratios, high-temperature areas are restricted to a narrow zone around the die opening. By contrast, large extrusion ratios extend the high-temperature zone inward into the billet and along the extruded product. Due to heat conduction toward the relatively cold die, the extrudate shows an obvious temperature gradient. A larger extrusion ratio aggravates temperature heterogeneity. Excessively high local temperature under an extrusion ratio of 26 may bring potential overheating risk. Overall, the extrusion ratio strongly governs thermal responses during hot extrusion: a larger extrusion ratio increases peak temperature, expands the high-temperature domain and exacerbates the temperature inhomogeneity of extruded parts.

**Figure 6:**
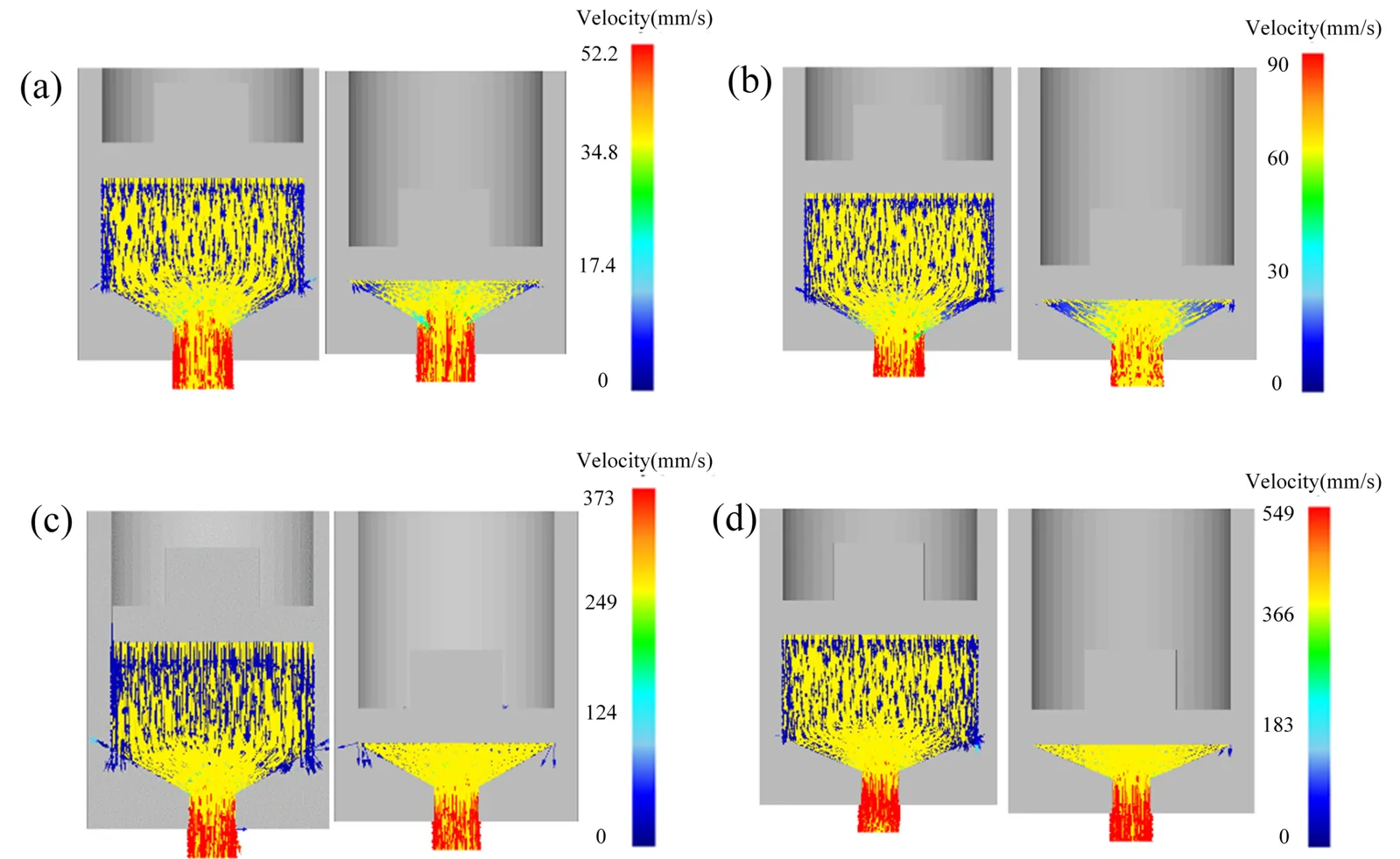
Flow field distributions during extrusions of 90 s/180 s with different extrusion ratios: (**a**) 13; (**b**) 16; (**c**) 20; (**d**) 26.

Figure 6a–d shows the contour maps of the velocity field distribution of 7A85 aluminum alloy at different moments during hot extrusion under extrusion ratios of 13, 16, 20 and 26. The vector arrows indicate material flow directions, and the colored contours represent velocity magnitude. At 90 s, material flows toward the die orifice. For all extrusion ratios, low velocity is observed in the bulk billet, while velocity increases drastically within the die deformation zone, with maximum velocity appearing in the extrudate at the die exit. Material flows radially toward the central axis before moving along the extrusion direction. Peak exit velocity rises remarkably with increasing extrusion ratio. The maximum velocity is 52.2 mm/s for an extrusion ratio of 13, and increases to 90 mm/s at an extrusion ratio of 16. As the extrusion ratio further increases to 20 and 26, peak velocity reaches 373 mm/s and 549 mm/s, respectively. According to the volume constancy principle, given a constant ram speed, a higher extrusion ratio corresponds to a smaller extrudate cross-section, which accounts for the sharp increase in exit flow velocity. At 180 s, steady-state extrusion is established and the overall flow feature is maintained. For low extrusion ratios of 13 and 16, the velocity gradient between the billet and extrudate is relatively mild. For large extrusion ratios (20 and 26), sharp velocity discontinuity occurs across the deformation zone. Frictional resistance from die tools slows down the material adjacent to the die surface, whereas the central material flows at much higher speed, producing obvious velocity discrepancy between the surface layer and core of the extrudate. A higher extrusion ratio also enhances the radial convergence of material within the die chamber. Such pronounced flow inhomogeneity at high extrusion ratios may induce inhomogeneous plastic deformation, thereby leading to microstructural inhomogeneity and residual stresses in the extruded product. Therefore, the extrusion ratio significantly affects the material flow characteristics. A higher extrusion ratio increases the maximum exit velocity, enlarges the internal velocity gradient, and aggravates flow inhomogeneity during hot extrusion.

### 4.2. Mechanism of Extrusion Ratio on Microstructure Evolution During Deformation

As a key parameter during the extrusion process, the extrusion ratio directly affected strain accumulation, temperature rise, and the competition between dynamic recovery and recrystallization by controlling the degree of deformation. Generally, increasing the extrusion ratio helps to break the original coarse structures, enhances the level of deformation energy storage, and is beneficial for dynamic recrystallization. However, most of the plastic deformation work during the extrusion process is converted into heat energy, and the larger the extrusion ratio, the more significant the deformation heat effect usually is. When the temperature rises to a certain level, dynamic recovery may occur preferentially, consuming part of the deformation energy, thereby partially inhibiting the full progress of recrystallization. Therefore, the influence of the extrusion ratio on the microstructure evolution is not a simple linear increase, but depends on the complex competition among deformation energy storage, the effect of rising temperature, and dynamic recovery. This study compared the grain size, misorientation angle, and texture characteristics of the as-extruded alloy under different extrusion ratios, revealing the intrinsic mechanism of the extrusion ratio on microstructure evolution.

**Figure 7:**
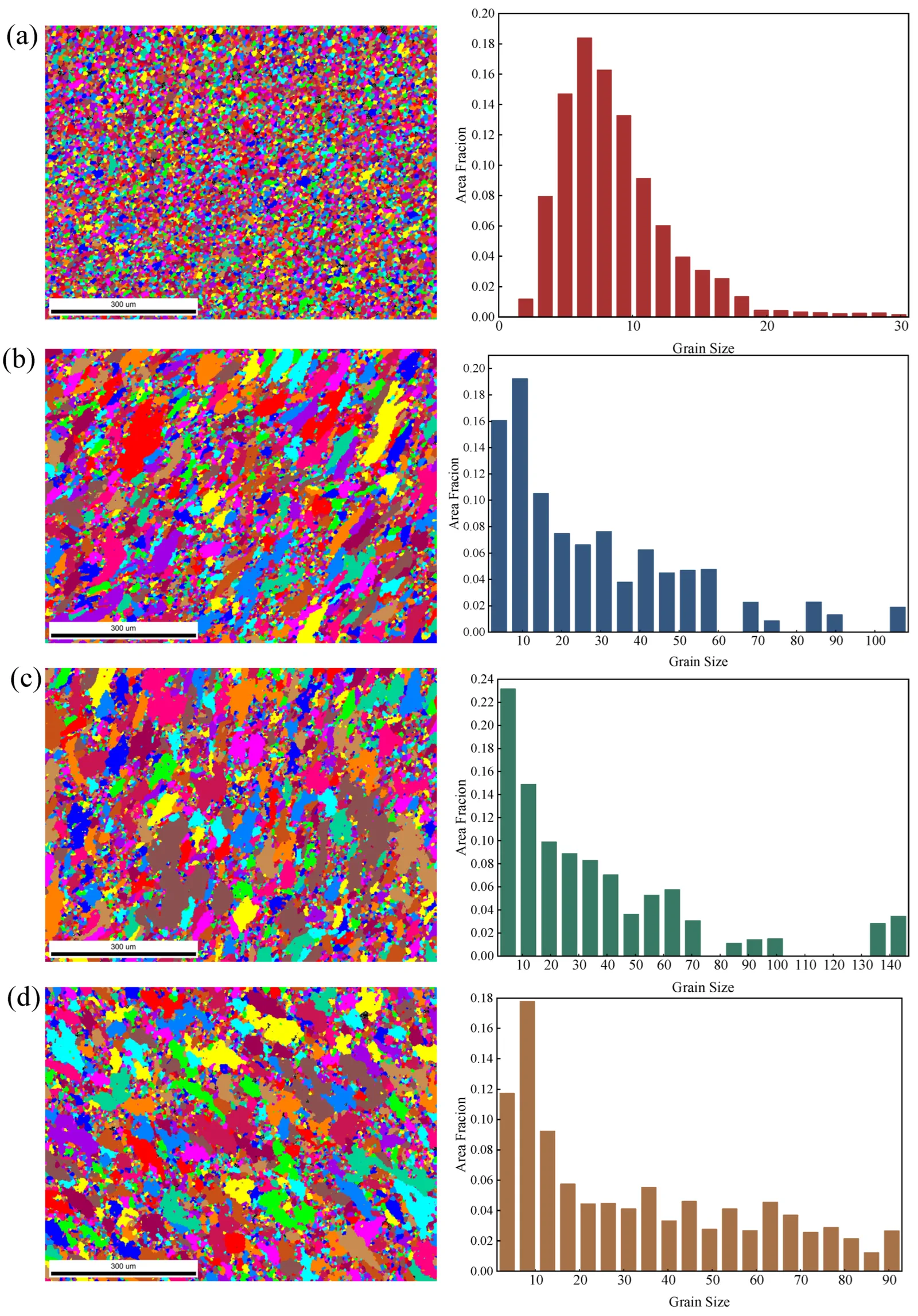
Grain size results under different extrusion ratios of (**a**) 13, (**b**) 16, (**c**) 20, and (**d**) 26.

Figure 7 shows the EBSD grain morphology images and corresponding grain size distributions of 7A85 aluminum alloy under different extrusion ratios. Overall, equiaxed grains were present in all the samples, indicating that different degrees of recrystallization occurred during the hot extrusion process. However, as the extrusion ratio changed, significant differences were observed in terms of grain size distribution and microstructure uniformity. At an extrusion ratio of 13, the alloy grains were fine and uniform, with almost no coarse grains observed, and the microstructure uniformity was the best. The grain size distribution was a narrow single-peak distribution, with the peak located at 5~10 μm, as shown in Figure 7a. This indicated that the recrystallization degree was relatively high at an extrusion ratio of 13, and grain growth was effectively inhibited, with the microstructure mainly consisting of uniform and fine equiaxed grains. Figure 7b showed the grain morphology characteristics and grain size distribution of the alloy at an extrusion ratio of 16. It can be seen that in addition to the fine equiaxed grains, a small number of coarse grains appeared, and locally, grains with a size exceeding 100 μm could be observed. The grain size distribution was significantly widened, and the microstructure uniformity significantly decreased. When the extrusion ratio was 20, fine grains and coarse grains coexisted, and the mixed grain characteristics were obvious. The number of coarse grains increased, and some grains could reach 120~145 μm in size. This indicated that at an extrusion ratio of 20, the microstructure was the most uneven; fine grains and coarse grains were mixed, suggesting incomplete recrystallization, and local areas may have experienced grain growth. Figure 7d showed the grain size distribution of the alloy at an extrusion ratio of 26, which was wider than that at 13. The number of coarse grains was significantly increased compared to 13, but the maximum grain size was smaller than that at 20. Under this condition, the grain size distribution was broadened, the microstructure uniformity was poor, and over-reduction and grain coarsening may have occurred.

After comprehensive comparison, as the extrusion ratio increased from 13 to 26, the average grain size of the alloy did not change significantly, remaining within the range of 5.7 to 6.5 μm. However, the distribution of grain sizes showed significant differences. The sample with an extrusion ratio of 13 had the narrowest distribution and the most uniform microstructure. Samples with extrusion ratios of 16, 20, and 26 all exhibited varying degrees of coarse grains, forming a mixed grain structure where fine and coarse grains coexist. Among them, the coarse grains in the sample with an extrusion ratio of 20 were the largest, and the mixed grain characteristics were the most obvious. These results indicated that the extrusion ratio significantly affected the uniformity of grain size and the degree of recrystallization in 7A85 aluminum alloy. It was worth noting that the average grain sizes of the three coarse-grain samples (with extrusion ratios of 16, 20, and 26) were comparable to that of the sample with an extrusion ratio of 13, all being around 6 μm, but their distribution patterns showed significant differences. This indicated that the average grain size alone could not fully describe the microstructure characteristics, and the distribution of grain sizes was also a key indicator for evaluating the integrity of recrystallization and the uniformity of the microstructure. Chen et al. [22] also reported a similar trend during the extrusion process of spray-formed Al-Zn-Mg-Cu alloy: when the extrusion ratio increased from 7 to 25, the average grain size remained basically unchanged (2~3 μm), while the content of coarse grains significantly increased. This was consistent with the results of this study and further indicated that in hot-extruded aluminum alloys, the sensitivity of the average grain size to changes in the extrusion ratio was relatively low, while the fraction and distribution pattern of coarse grains had a more significant impact on the uniformity of the microstructure.

**Figure 8:**
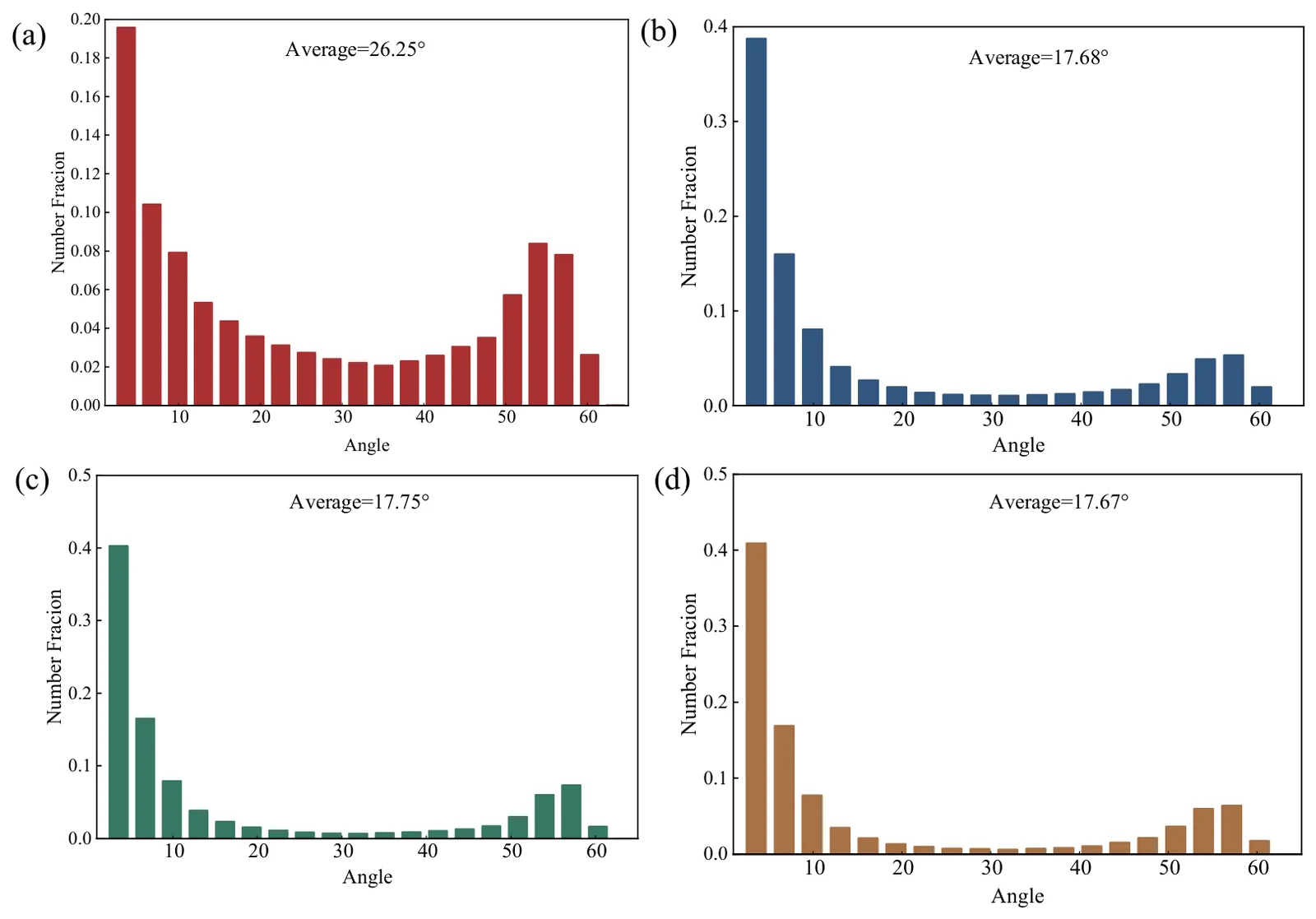
Misorientation angle results under different extrusion ratios of (**a**) 13, (**b**) 16, (**c**) 20, and (**d**) 26.

Figure 8 showed the distribution of misorientation angles for 7A85 aluminum alloy under different extrusion ratios. The grain size reflected the degree of refinement of recrystallized grains, while the misorientation angle distribution provided further evidence for the degree of recrystallization through the relative proportions of low-angle grain boundaries (LAGBs, 2–15°) and high-angle grain boundaries (HAGBs, >15°). When the extrusion ratio was 13, the average misorientation angle was the highest (26.25°), with a wide and continuous distribution dominated by high-angle grain boundaries, as shown in Figure 8a. Combined with the most uniform grain size characteristics in Figure 7, this indicated that the degree of recrystallization was relatively high under this condition. In contrast, for samples with extrusion ratios of 16, 20, and 26, the average misorientation angles were 17.68°, 17.75°, and 17.67°, respectively, which were significantly lower and at similar levels. More importantly, the misorientation angle distributions of these samples still retained obvious LAGB peaks, indicating that a considerable amount of deformation substructures (such as subgrain boundaries) were retained, and the degree of recrystallization was relatively low [12,23,24,25]. Sun et al. [26] found in AA7050 aluminum alloy extrusions that although a higher extrusion ratio increased the stored deformation energy, a significant temperature increase might lead to dynamic recovery effects exceeding recrystallization, resulting in a higher proportion of LAGB peaks. When the extrusion ratio increased to 16 and 20, although the deformation degree was greater than that of the sample with an extrusion ratio of 13, the degree of recrystallization was not as high as the latter, indicating that when the extrusion ratio was large, incomplete recrystallization was not only caused by insufficient stored energy, but might also be related to the promotion of dynamic recovery by the increase in deformation temperature and the premature consumption of recrystallization driving force. As the extrusion ratio increased from 13 to 16 and 20, the increase in stored energy caused by strain and the acceleration of dynamic recovery caused by the increase in deformation temperature formed a competition. On the one hand, some dislocations were consumed by dynamic recovery before recrystallization nucleation. On the other hand, a certain amount of substructures were still retained. When the extrusion ratio was 26, the average misorientation angle (17.67°) was similar to those of 16 and 20, indicating that the substructures were still retained to a certain extent.

**Figure 9:**
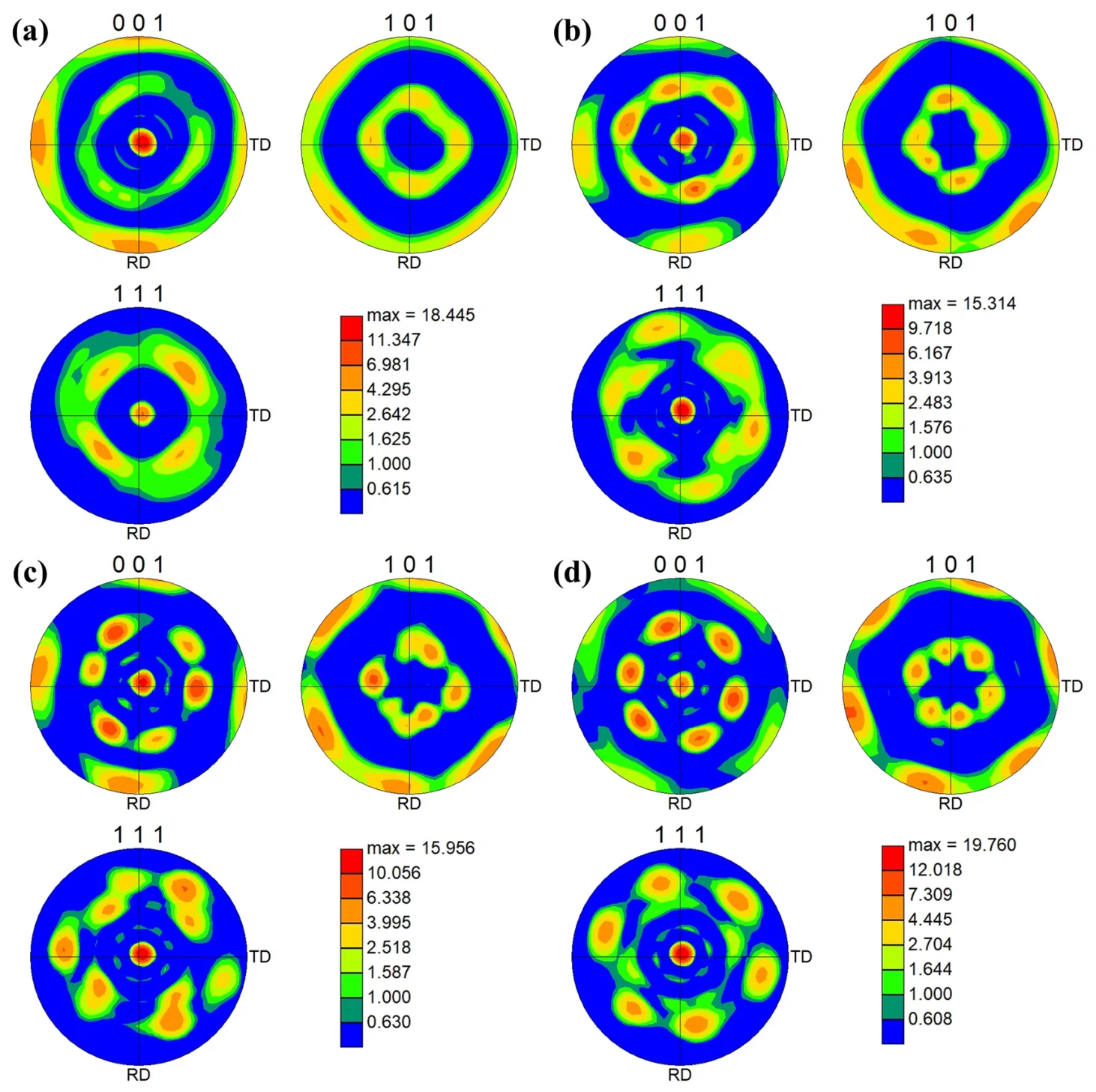
The pole figures under different extrusion ratios of (**a**) 13, (**b**) 16, (**c**) 20, and (**d**) 26.

Figure 9 shows the texture diagrams of 7A85 aluminum alloy with different extrusion ratios. The maximum texture strength of the alloy under extrusion ratios of 13, 16, 20, and 26 was 18.45, 15.31, 15.96, and 19.76, respectively. At each extrusion ratio, the alloy exhibited a strong deformation texture. It was notable that although the recrystallization degree at an extrusion ratio of 13 was relatively high, its texture strength was still higher than those at extrusion ratios of 16 and 20, indicating that dynamic recrystallization did not completely randomize the grain orientation. The recrystallized grains might have inherited part of the deformation texture. The extrusion ratio of 26 showed the highest texture strength. Combined with its relatively wide grain size distribution, this indicated that the deformation texture was retained relatively intact and recrystallization was strongly inhibited. This might have been related to the fact that the excessively high extrusion ratio caused the temperature to rise above a certain threshold, and dynamic recovery replaced recrystallization as the main softening mechanism. The stored deformation energy was consumed in advance, which allowed the deformation texture to be retained.

Combining the EBSD analysis with the tensile properties of the as-extruded samples, the extrusion ratio of 20 showed incomplete recrystallization, retained a relatively high fraction of LAGBs, and exhibited a distinct mixed grain structure. The coarse grains could reach 120~145 μm, but the as-extruded tensile strength was the highest (502 MPa). This phenomenon might have been related to the combined effect of grain-boundary strengthening and substructure retention (such as LAGBs). At an extrusion ratio of 13, the degree of recrystallization was relatively high, and fewer substructures such as LAGBs were retained. Although the contribution of grain-boundary strengthening was significant, the overall strength did not reach the highest value. At an extrusion ratio of 20, due to incomplete recrystallization, a relatively higher density of substructures (with a higher proportion of LAGBs) might have been retained, thereby contributing to the strength. Ni et al. [27] also observed a similar phenomenon when studying extruded rods of the Al-Mg-Si-Cu alloy. When the extrusion ratio increased and recrystallization was inhibited, a higher density of substructures could lead to an increase in strength. At an extrusion ratio of 26, although substructures were still retained to some extent, the coarse-grain characteristics were further exacerbated, the grain size distribution was broader, reaching up to 90 μm, and the texture strength was the highest (19.76), indicating more pronounced deformation inhomogeneity. This inhomogeneity might have caused local stress concentration and led to plastic instability, resulting in a slightly lower strength than that at an extrusion ratio of 20 (491 MPa). However, it should be noted that this study did not quantitatively characterize the dislocation density or substructure fraction, but only made qualitative inferences based on the grain morphology, grain size distribution, and misorientation distribution obtained from EBSD. Overall, the strength of the extruded alloy was influenced by the combined effects of grain-boundary strengthening and substructure strengthening (such as LAGBs), and the relative effect of the two depended on the balance between the degree of recrystallization and the retention of substructures. In applications that prioritized the as-extruded strength, retaining some substructures rather than achieving a higher degree of recrystallization might have been more effective. In subsequent heat treatment processes, an evenly fine-grained microstructure was more conducive to obtaining a stable aging strengthening response.

## 5. Conclusions

(1) The tensile properties of the as-extruded alloy showed a trend of increasing first and then decreasing with the increase in the extrusion ratio. The best strength combination (tensile strength of 502 MPa, yield strength of 348 MPa, elongation of 10.23%) was obtained when the extrusion ratio was 20. When the extrusion ratio increased to 26, the strength decreased and the elongation increased to 11.47%. With the increase in the extrusion ratio, the hardness of the extruded alloy increased from 128 HBW to 135 HBW, and the electrical conductivity decreased from 35.64% IACS to 33.79% IACS.

(2) The tensile properties, hardness, and electrical conductivity of the T6-treated alloy were relatively insensitive to the extrusion ratio. Within the extrusion ratio range of 13 to 26, the tensile strength remained stable at 689 to 697 MPa, the yield strength remained stable at 650 to 655 MPa, the hardness remained stable at 177 to 182 HBW, and the electrical conductivity remained stable at 32.1 to 32.3 %IACS.

(3) When the extrusion ratio was 13, a uniform equiaxed recrystallization structure was obtained, with an average grain size of 5.73 μm and an average misorientation angle of 26.25°. When the extrusion ratio was 16, 20, and 26, the temperature increase caused by deformation heating accelerated dynamic recovery and to some extent promoted the growth of recrystallization grains, forming a mixed grain structure composed of fine grains and coarse grains. Strong deformation textures (texture intensity: 15.3~19.8) were observed in all four extrusion ratios, and the orientations of the recrystallization grains were close to that of the matrix.

(4) Considering microstructure homogeneity and mechanical properties comprehensively, an extrusion ratio of 13 is preferred for optimal microstructural uniformity, while an extrusion ratio of 20 yields the highest as-extruded strength. The practical process parameters should be balanced according to targeted service performance requirements.

## Data Availability

The original contributions presented in this study are included in the article. Further inquiries can be directed to the corresponding author.
